# Zinc, Copper, and Iron in Oral Submucous Fibrosis: A Meta-Analysis

**DOI:** 10.1155/2018/3472087

**Published:** 2018-06-26

**Authors:** Prageet K. Sachdev, Jeanne Freeland-Graves, S. Natasha Beretvas, Namrata Sanjeevi

**Affiliations:** ^1^Department of Nutritional Sciences, College of Natural Sciences, The University of Texas at Austin, 103 W. 24th St. A2703, Austin, TX 78712, USA; ^2^Department of Educational Psychology, The University of Texas at Austin, George I. Sánchez Building 538E, 1912 Speedway, Austin, TX 78712, USA

## Abstract

Oral submucous fibrosis (OSF) is a potentially malignant disorder which causes fibrosis and inflammation of the oral mucosa. Studies have reported altered levels of trace elements in oral submucous fibrosis subjects, but findings have been inconsistent. The objective of this research is to perform a meta-analysis to summarize studies that report zinc (Zn), copper (Cu), and iron (Fe) in patients, with and without OSF. A literature search of Embase, PubMed, Cochrane Library, and Web of Science electronic databases was conducted for studies up to January 2017. A total of 34 reports met the inclusion criteria. The standardized mean difference was utilized as the effect size. The robust variance estimation method was chosen to handle dependency of multiple related outcomes in meta-analysis. There was a significant increase in the levels of Cu (effect size = 1.17, *p* value < 0.05, 95% confidence interval (CI): 0.164–2.171) and a significant decrease in levels of Zn (effect size = −1.95, *p* value < 0.05, 95% CI: −3.524 to −0.367) and Fe (effect size = −2.77, *p* value < 0.01, 95% CI: −4.126 to −1.406) in OSF patients. The estimation of Zn, Cu, and Fe levels may serve as additional biomarkers in the diagnosis and prognosis of OSF along with the clinical features.

## 1. Introduction

Oral submucous fibrosis (OSF) is a potentially malignant disorder (WHO, 2017) that causes fibrosis and inflammation of the oral mucosa [1]. This condition is more prevalent in South and Southeast Asia, particularly India, with fewer cases reported in Europe and North America [2–4]. The prevalence of OSF cases has increased from 0.03% to 6.42% in the last four decades, making it a significant public health problem in India [3]. This disease contributes significantly to mortality because of its high malignant transformation rate of 7.6% [4, 5]. The disease is more prevalent in men, perhaps due to taboos associated with oral chewing habits in women [4]. Several studies have provided evidence that chewing of areca nut is the primary etiological factor for the development of OSF [6–9]. In a case-control study, it was found that 98% of the patients with OSF chewed areca nut regularly, compared to 38% among the healthy controls [7]. Maher et al. observed an increased risk of development of OSF in those who chewed areca nut (relative risk = 154) [8].

The areca nut grows on the areca catechu palm tree which is commonly found in Pacific Islands and South Asia [10]. It is consumed wrapped in betel leaves or in form of commercial preparation of pan masala, mawa, gutkha (a preparation of betel nuts and tobacco designed to be chewed), or supari [10]. The areca nut is composed of alkaloid components [10]. The most potent alkaloid, arecoline, causes an abnormal increase in the production of collagen by the oral mucosal fibroblasts. This over production leads to the development of fibrotic bands in the buccal mucosa of OSF patients [11]. The condition begins as an inflammatory response to areca nut chewing, followed by fibrosis of the oral submucosa and then progresses to induce restriction of the mouth opening and difficulty in mastication and swallowing [10]. The early stage symptoms include burning sensation in the mouth, development of ulcers and vesicles, increased salivation, and blanching of the mucosa. In the later stages, the mucosa becomes inelastic and leathery because of the fibrotic bands [7, 10].

Altered trace element status has been reported in both the potentially malignant and malignant stages [12–14]. Microminerals are well established to be essential in metabolism as components of enzymes and hormones in the body. This research will focus on three trace minerals—Zn, Cu, and Fe—that are altered in OSF. Zinc is crucial for the normal functioning of the immune cells [15], antioxidant defense, wound healing, and stability of biological membranes [15]. Chewing of areca nut generates reactive oxygen species, which can cause damage to the proteins and nucleic acids in the body. Zn induces activation of antioxidant enzyme superoxide dismutase (SOD) which inhibits production of reactive oxygen species [15]. The levels of SOD were found to be reduced (75.42 ± 7.04) in OSF patients as compared to controls (177.17 ± 5.92) [16]. The mineral copper plays an important role in the formation of red blood cells and synthesis of collagen in the bones and connective tissue and aids in the absorption of iron [17]. It is also essential for the functioning of enzymes such as Cu/Zn-superoxide dismutase and lysyl oxidase [18]. Related to OSF, Cu is a cofactor for lysyl oxidase, the enzyme involved in the maturation of collagen [19]. The areca nut contains a high content of Cu that is released within 5–30 minutes of chewing the nut, inducing upregulation of lysyl oxidase in the oral mucosa [20, 21]. Trivedy et al. observed that the levels of Cu in the saliva peaked after 10 mins in two volunteers and 20 minutes in the third volunteer after chewing an areca nut product for 30 minutes [20]. It has been suggested that the elevated levels may then induce excessive collagen formation by the fibroblasts [21]. Iron is required for the functioning of numerous enzymes such as cytochrome oxidase, xanthine oxidases, succinate dehydrogenase, glucose-6-phosphate dehydrogenase, catalases, peroxidases, and choline dehydrogenase [22]. It acts as a cofactor for prolyl hydroxylase (PH) and lysyl hydroxylase, which are enzymes involved in hydroxylation of collagen [22]. Excessive collagen synthesis in OSF may result in a decline in the levels of serum and plasma Fe in OSF patients. Finally, the burning sensation and the restricted mouth opening in this disease limits the intake of food, further exacerbating deficiencies of micronutrients [23, 24]. The Fe dependent enzyme, cytochrome oxidase, is required for the development of the epithelium [25]. A deficiency of iron in OSF reduces the levels of cytochrome oxidase, resulting in atrophy of the epithelium [25]. Investigations have reported diminished Zn and Fe values in OSF subjects, as compared to healthy controls [26–31]. In contrast, Cu levels in serum/plasma/saliva have been observed to be elevated rather consistently [23, 26, 28–30, 32–38].

Although studies have attempted to explore the status of trace elements in OSF patients, results to date have been inconclusive. Differences exist in sample age, gender, biological samples (serum, plasma, and saliva), and laboratory methods used by the investigators. In addition, all these studies have measured multiple micronutrients (outcomes), which may have synergistic and antagonistic interactions. Thus, multiple outcomes reported by each study cannot be considered independent of each other. In order to adjust for these interactions in calculation of the pooled effect sizes for each micronutrient, information on correlations is needed from each study. However, the primary studies did not report correlations between the trace elements. Consequently, this research will use a sophisticated statistical technique of robust variance estimation to (a) summarize the existing literature and conduct a meta-analysis to evaluate differences in Zn, Cu, and Fe status between OSF and healthy participants and (b) identify the influence of moderators (male sex and biological samples (plasma, serum, or saliva)). The robust variance estimation is a fairly new and powerful approach to handle dependency between multiple nonindependent outcomes when the information on within-study covariance is not available [39]. In addition, this research also will calculate ratios of Zn/Cu and Cu/Fe from the primary studies; these will be used further to generate a weight average or pooled difference in these ratios between OSF and healthy participants.

## 2. Methods and Materials

### 2.1. Data Sources and Search Strategy

**Figure 1:**
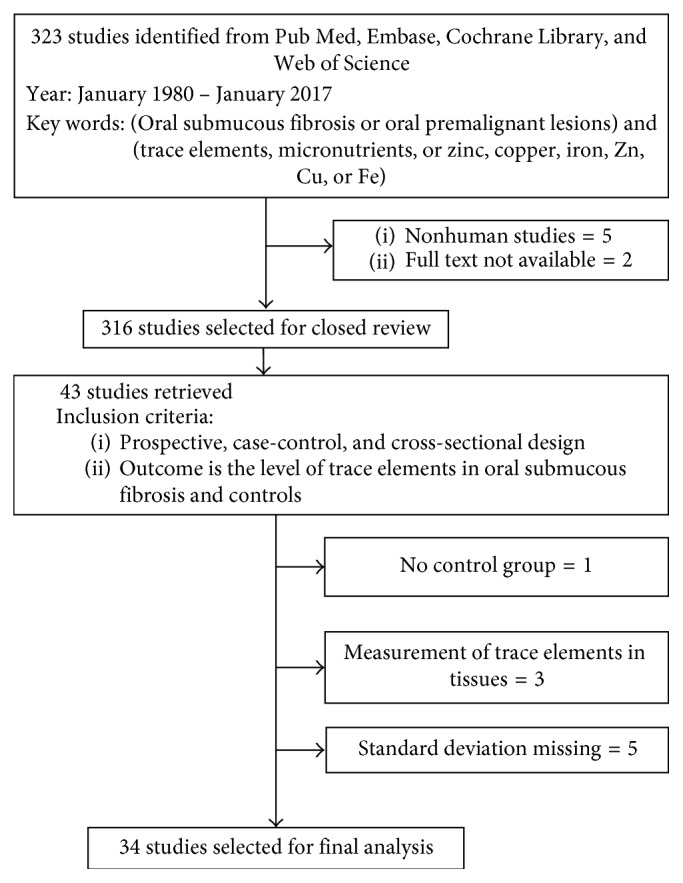
Flowchart illustrating the process of study selection for the meta-analysis.

A comprehensive literature search was conducted to identify relevant prospective, case-controlled, and cross-sectional studies investigating the status of Zn, Cu, and Fe in OSF from 1980 to January 2017. The electronic databases of PubMed, Embase, Web of Science, and Cochrane Library were searched as detailed in Figure 1. The reference list of selected articles and reviews on the topic also were searched. A total of 323 studies were extracted, using the keywords oral submucous fibrosis, oral potentially malignant lesion and trace elements, micronutrients, zinc, copper, iron, Zn, Cu, and Fe. Seven studies were excluded because of nonavailability of full-text articles or nonhuman research articles. The 42 articles finally retrieved were then subject to the exclusion criteria, resulting in a total of 34 studies that were used for the final analysis.

### 2.2. Inclusion and Exclusion Criteria

All potentially relevant publications were reviewed independently by two reviewers using the inclusion criteria. Inclusion criteria were prospective, cross-sectional, or case-control investigations and studies measuring concentrations of trace elements in both healthy controls and OSF participants.

Exclusion criteria were studies with inadequate information, such as sample size and standard deviation for the calculation of the effect sizes, absence of the control group, measurement of tissue levels of the micronutrients, and nonhuman research. Duplicate experiments were removed, and any disagreement regarding inclusion was resolved with the aid of a third reviewer. Five studies that did not report sample size and standard deviation were excluded [40–44]. Also, two studies that measured only tissue levels of micronutrients [45–47] and one study that did not have a control group were excluded [46].

### 2.3. Data Extraction

Mean values and standard deviations of plasma, serum, and salivary Zn, Cu, and Fe in control and OSF groups were extracted from the included studies. Author, the year of publication, sample size, and age of participants also were recorded. In addition, the type of biomarker (serum/plasma/saliva) and proportion of males among the OSF participants were collected to conduct moderator analysis. This meta-analysis included 34 studies, with total of 2409 participants.

### 2.4. Statistical Analyses

All data were analyzed in the Statistical Package for Social Sciences (SPSS Version 22, Armonk, NY, 2013) [48]. Characteristics of the publications that were included are presented in Table 1 [12, 23, 24, 26–38, 49–66]. Standard deviations of the micronutrient values and their associated sample sizes were used to calculate the pooled sample variances. The standardized mean differences, Hedges *g* [67], were calculated by subtracting the means of the trace element values in the healthy controls from the means of OSF patients and then dividing the difference by the pooled standard deviation. The above procedure was performed to handle the different units of measurement used. Small-sample bias correction was applied to obtain the bias-corrected Hedges *g*. The variance and 95% confidence interval estimate of the effect size estimates were determined.

The negative effect sizes represented lower levels of the trace elements in the OSF participants, as compared to the healthy controls. More than one effect size was extracted from each report to explore interactions among the minerals in the disease.

Studies included in a meta-analysis usually contain information on only one outcome. The present report differs in that each investigation provides levels of three trace elements, Zn, Cu, and Fe (three outcomes) from the same individuals; this results in effect sizes that are correlated. Two possible approaches to handle the multiple outcome data are (1) to incorporate correlations between the micronutrients as reported by the original studies in the analysis or (2) discard effect sizes such that each study contributes only one effect size but with the downside of losing information on some of the micronutrients [39]. The original studies on OSF did not provide correlations between these three trace elements. In addition, previous investigations have observed that increased Cu is associated with a reduction in Zn, thereby implying that these effect sizes are not independent of each other [23, 29]. When the dependence between multiple outcomes is not taken into consideration, the assumed correlation is 0, which implies that the outcomes are unrelated. This results in an underestimation of variance of the overall effect size. In contrast, a correlation of 1 creates an overestimation of the variance for the composite effect size. Both these scenarios can inflate Type I and Type II errors, respectively [68]. In this research, the pooled average effect size and variance were calculated using the robust variance estimation method, by applying Tipton's [69] small-sample bias correction in the statistical software R (3.4.0). The robust variance estimation (RVE) is a sophisticated meta-analytical technique that handles dependency between outcomes, while controlling for Type I and Type II errors [39]. It does not require the information on the within-study covariance of the original studies and is not based on the assumption of normality of the effect sizes [39, 68]. The output included the pooled average effect size, the standard deviation, the Z-statistic, and 95% confidence intervals. The effects of the moderators on the effect size were analyzed using the mixed effects meta-regression model. All the results were tested using an alpha level of 0.05 to discern statistical significance. Forest plots were created to graphically represent the results of the meta-analysis. All the equations and formulas used in the analysis were derived from *The Handbook of Research Synthesis and Meta-Analysis* [68].

The ratios of Zn/Cu and Cu/Fe also were calculated from all studies (Tables 2 and 3 resp.). All studies had different sample sizes, so a weighted average or pooled estimate of the difference in ratios of levels of trace elements between OSF and controls was calculated. This provided more weight to studies that had a larger sample size. Thus, the weighted average of the differences between Zn/Cu ratios in OSF and healthy controls was determined. Similarly, the weighted average of difference between Cu/Fe ratios in OSF and healthy controls also was determined via the following formula:

(1) $$\begin{array}{c} \left[\left(n1\;*\text{ difference}\;\;\mathrm{in}\;\;\text{Zn}/\text{Cu}\left(\text{OSF}-\text{controls}\right)\right)+\left(n2\;*\text{ difference}\;\;\mathrm{in}\;\;\text{Zn}/\text{Cu}\left(\text{OSF}-\text{controls}\right)\right)+\left(n3\;*\text{ difference}\;\;\mathrm{in}\;\;\text{Zn}/\text{Cu}\left(\text{OSF}-\text{controls}\right)\right)+\left(n\;*\text{ last}\;\;\mathrm{difference}\;\;\mathrm{in}\;\;\text{Zn}/\text{Cu}\left(\text{OSF}-\text{controls}\right)\right)\right]\cdot \left(n1+n2+\cdots +n\left(\text{last}\;\;\mathrm{study}\right)\right), \end{array}$$

where *n*  is the total sample size of a study.

## 3. Results

Figure 1 illustrates the steps of the screening process involved in the selection of articles for the meta-analysis. The numbers of effect sizes for Zn, Cu, and Fe were 17, 24, and 23, respectively, for a total of 64 effect sizes. There was a significant increase in the levels of Cu (effect size = 1.17, *p* value < 0.05, 95% confidence interval (CI): 0.164–2.171) and a significant decrease in levels of Zn (effect size = −1.95, *p* value < 0.05, 95% CI: −3.524 to −0.367) and Fe (effect size = −2.77 *p* value < 0.01, 95% (CI): −4.126 to −1.406) in OSF patients, as compared to controls. However, the high I^2^ value of 97.24% indicates a high level of heterogeneity between the studies included.

The pooled differences in Zn/Cu ratio and Cu/Fe ratios between OSF and healthy participants were −0.3046 and 0.9826.

### 3.1. Forest Plots

**Figure 2:**
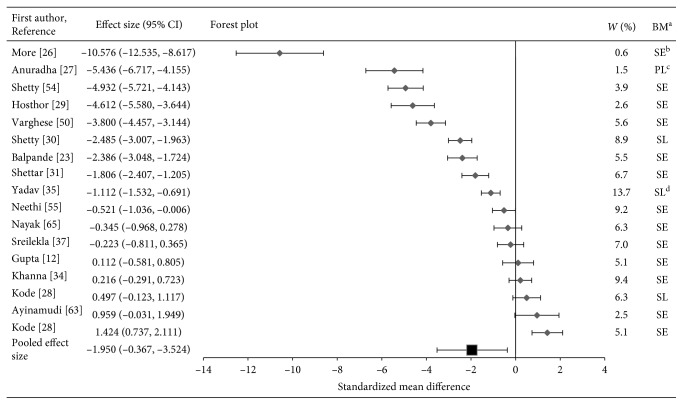
Forest plot of pooled effect size estimates and 95% confidence intervals representing differences in levels of salivary, serum, or plasma zinc between oral submucous fibrosis patients and healthy controls.

**Figure 3:**
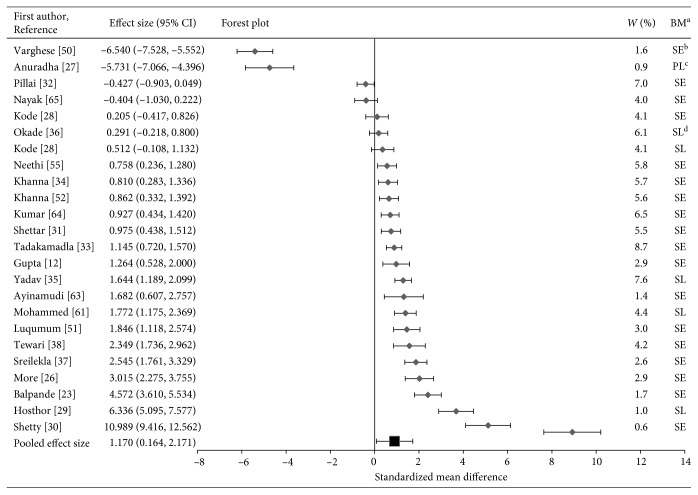
Forest plot of pooled effect size estimates and 95% confidence intervals representing differences in levels of salivary, serum, or plasma copper between oral submucous fibrosis patients and healthy controls.

**Figure 4:**
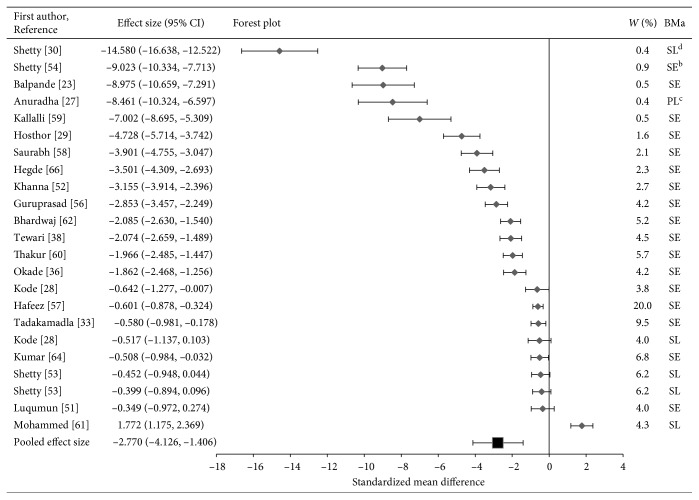
Forest plot of pooled effect size estimates and 95% confidence intervals representing differences in levels of salivary, serum, or plasma iron between oral submucous fibrosis patients and healthy controls.

The results on levels of Zn and Fe in OSF versus healthy participants in this meta-analysis are supported further by the forest plots in Figures 2–4. The majority of the first studies (*n*=10) in the forest plot for Zn show lower zinc levels in OSF subjects as compared to healthy participants. The results from five studies were not statistically significant [4, 12, 28, 34, 37] since the confidence intervals crossed the line of null effect (standard mean difference = 0). Two studies showed high Zn levels in OSF patients [28, 63].

In the forest plot for Cu (Figure 3), the majority of the studies (*n*=18) illustrate the higher levels of Cu in OSF patients as compared to controls. Two studies [27, 50] showed exceptionally low levels of copper. Yet, the results of five studies were not statistically significant [4, 28, 32, 36] since the confidence interval crossed the line of null effect.

Finally, in the forest plot for iron, majority of the studies (*n*=21) showed lower levels of Fe in OSF patients as compared to that of healthy controls. However, one study documented higher Fe levels in OSF patients [61] as compared to the controls.

It should be noted that the two studies that had large effect sizes and suggested outliers were removed from the analysis [24, 49]. The horizontal axis (*x*-axis) of forest plots represents the standardized mean differences. The vertical line in the plot indicates “line of null effect” or no statistically significant difference in levels of trace elements between the OSF and control groups. The average effect sizes for Zn, Cu, and Fe are illustrated by the square symbol in the bottom most row in the respective forest plots. The horizontal line passing the square symbol demonstrates a 95% confidence interval for the average effect size. Each diamond symbol in the forest plot indicates the effect size for a study. The experiments with a greater number of participants have lower confidence intervals.

### 3.2. Moderator Analysis

The biomarkers (saliva/serum/plasma) and proportion of men in the OSF group were used as moderators. The subgroup analysis indicated that none of the moderators had a significant influence on the effect size (*p* > 0.05). Although it is known that a greater proportion of men than women suffer from OSF, the statistically insignificant results for the moderator could be due to the smaller number of investigations that reported the sex of participants.

## 4. Discussion

This is the first meta-analysis to collectively analyze the levels of trace elements of Zn, Cu, and Fe in OSF participants as compared to healthy controls, controlling for the interactions between these micronutrients. This meta-analysis made use of the sophisticated statistical technique of robust variance estimation to handle the dependency between trace elements. The results of this research suggest that the levels of Zn and Fe are lower, and Cu levels are higher, in participants with OSF. The high extent of heterogeneity between the studies, as seen by the high I square value, could be due to the varying type of biological samples (serum, plasma, or saliva) and/or the sex of the participants. The gender and biological samples (serum, plasma, or saliva) used was included as moderator to evaluate for any discrepancies in levels of micronutrients affecting the overall results. Both the biological sample and gender did not affect the difference in the levels of trace elements between OSF and healthy controls. The level of plasma/serum/saliva Zn in OSF participants has been evaluated in numerous investigations [4, 23, 26, 27, 29–31, 35, 37, 55]. Only four of these did not report lower Zn levels in OSF patients [12, 28, 34, 63].

An imbalance in the ratio of Zn to Cu has been observed in malignant [70, 71] as well as inflammatory conditions [72]. Dietary Zn interferes with Cu absorption by inducing the synthesis of metallothionein, a protein which sequesters copper, making it unavailable for absorption [73]. Shettar [31] has documented a progressive increase in serum copper levels from Grade I (126 *μ*g/dl) to Grade IV (146 *μ*g/dl) of OSF, with an increase in Cu/Zn ratio with advancement of the disease. Fe has been observed to interfere in the absorption of Cu in the blood [74]. The ratios of Zn/Cu and Cu/Fe were also calculated from all the studies in order to identify whether a common trend indicating alterations in these ratios exits in OSF patients as compared to healthy participants. A decrease in the ratio of Zn/Cu (Table 2) was documented suggesting a decline in Zn, increase in Cu levels, or both in OSF patients as compared to controls. Similarly, an increase in the Cu/Fe ratio can be observed (Table 3), suggesting an increase in Cu, decrease in Fe, or both in OSF patients as compared to controls. The pooled differences in Zn/Cu ratio and Cu/Fe ratios between OSF and healthy participants were −0.3046 and 0.9826. These numbers indicate that the pooled difference in Zn/Cu ratio was negative and Cu/Fe was positive between OSF and healthy participants, which may reflect the underlying oxidative stress status. Increase in Cu can exacerbate the oxidative stress in the body by elevating the free-radical production. Alterations in these ratios indicate a disease status but may also be beneficial in indicating any advancement in the diseases towards malignancy. These results also stress the use of copper chelation therapies, which reduce the levels of Cu in OSF participants.

Many reasons have been suggested to explain the decreased Zn levels in OSF participants. This trace mineral is known to be an important antioxidant via several mechanisms. First, it acts as the cofactor Cu/Zn-superoxide dismutase enzyme (Cu/Zn-SOD) to serve as the first-line of defense against reactive oxygen species in the cells [75]. Second, it is crucial for the gene expression of the antioxidant protein, metallothionein, which scavenges hydroxyl ions and protects the cells against oxidative damage [76]. Third, zinc competes with the transition metals for binding sites, reducing the availability of those metals for reactions that generate hydroxyl ions [76]. Neutralization of free radicals generated by the areca nut may cause excessive cellular uptake of Zn, thereby resulting in decreased Zn levels in the patients [15].

Zn also may play an important role in preventing the development of malignancy. The Zn-dependent protein p-53 gene is involved in the repair of DNA [76]. In addition, the transcription factors of AP-1 and NF-*k*B which regulate apoptosis and defense responses against oxidative stress can undergo alterations with the reduction in cellular levels of zinc [76]. Thus, deficiency of zinc may disrupt the processes of DNA repair, apoptosis, and increase the susceptibility of the cells to oxidative stress [76]. A lack of Zn also induces overexpression of COX-2 which may promote cell proliferation and inhibit apoptosis, thereby contributing to the malignant transformation of OSF to oral carcinomas [76].

In contrast, relatively fewer studies have documented *higher* Zn concentrations in OSF [28, 63], as compared to healthy subjects. Although Khanna et al. [34] reported greater Zn levels in OSF subjects, differences were not statistically significant. The consumption of gutkha (a preparation of betel nut), which has a high Zn content, could possibly explain the elevated zinc levels in some OSF subjects. Several publications have studied the effect of Zn supplementation in diseases similar to OSF that are induced by free-radical damage such as type I diabetes [77] and macular degeneration [78].

Copper (Cu) is an integral part of Cu/Zn-SOD, an enzyme which also serves as an important antioxidant defense mechanism in the body. The SOD enzyme changes the superoxide ion O_2_^−^ into either molecular oxygen (O_2_) or hydrogen peroxide (H_2_O_2_) by either addition or removal of an electron [18]. In this process, there is either a reduction of the Cu^2+^ to Cu+ or oxidation from Cu+ to Cu^2+^ state [18]. Yet, when Cu is present in high concentrations, it generates reactive oxygen species that induce oxidative damage to the cell [79, 80]. Several studies have reported elevated Cu levels in the sera of OSF patients [31, 33, 63], presumably due to chewing areca nut which is rich in Cu (302 nmol/gm) [49]. High levels of Cu in the serum of OSF patients also may be attributed to the inflammatory response to the areca nut, in which the liver releases the copper-carrying ceruloplasmin protein [63]. Finally, decreased catabolism of ceruloplasmin may increase Cu levels in OSF patients [63]. Though there is not enough evidence on the release of copper from its enzymes in OSF, Winyard et al. observed that ceruloplasmin releases Cu during the oxidative stress induced by low-intensity UV irradiation [81]. OSF induces a state of oxidative stress, where ceruloplasmin may be releasing increased Cu in the body.

The chewing of the areca nut releases Cu which is taken into the oral mucosal keratinocytes through a non-enzyme-dependent diffusion process, bound to the protein metallothionein [8]. The increased copper upregulates the activity of lysyl oxidase, which increases collagen production. Several studies have investigated the copper levels in fibrotic lesions. Trivedy et al. documented upregulation of collagen synthesis by oral fibroblasts that were incubated with copper chloride as compared to those without copper [46]. Neve et al. documented significantly higher mean copper levels in the erythrocytes, but not in plasma, in cystic fibrosis patients (3.56 *μ*g/g Hb ± 0.50) as compared to healthy age-matched controls (2.73 *μ*g/g Hb ± 0.30) [82]. Also, reduced activity of two copper containing enzymes, cytochrome oxidase and Zn–Cu SOD, was documented in the neutrophils and erythrocytes of children suffering from cystic fibrosis [83]. Increased hepatic copper was observed in cirrhotics, with a significant association between the excess copper and liver fibrosis [84]. Although increased iron overload also promotes collagen synthesis, it appears to be more closely related to steatosis than to fibrosis in chronic alcoholics [85]. In contrast, Hughes et al. found no significant difference in levels of serum copper and ceruloplasmin between controls and patients suffering from systemic sclerosis [86]. The effectiveness of copper chelators in treatment of fibrotic lesions also has been established in animal models [87, 88]. Brewer et al. documented inhibition of fibrosis (independent of inhibition of inflammation) in the bleomycin mouse model of pulmonary fibrosis treated with tetrathiomolybdate, a copper chelator [87]. Finally, Gong et al. observed amelioration in diabetes-evoked renal fibrosis in rat cells treated with a copper chelator [88].

The role of excess copper in carcinogenesis also has been the subject of investigations [89–91]. High intracellular levels of copper can generate hydroxyl radicals that cause damage to the DNA and protein molecules. In addition, this may activate angiogenesis factors such as tumor necrosis factor alpha and vascular endothelial growth factor [89]. These angiogenetic factors play an important role in tumor growth and metastasis [89]. The blood level of ceruloplasmin (a Cu-transporting protein) has been observed to be increased from four- to eightfold during OSF malignancy [13]. Ceruloplasmin serves as a source of Cu(I) ions, which may initiate the process of LDL oxidation and play a role in the malignant transformation of OSF to oral carcinoma [91]. The high copper content can generate reactive oxygen species by Fenton and Haber–Weiss reaction [92].

Two publications reported statistically significant lower levels of Cu in OSF participants [27, 50] as compared to healthy controls. Varghese et al. suggested that the decrease in copper levels noted in the study, contrary to prior studies, could be due to the difference in lab methodologies employed and the selection of the patients [50]. This study used atomic absorption spectrophotometry to measure levels of copper, and the patients selected were not on any treatments as opposed to earlier studies that used calorimeter and the patients were on treatment for OSF. However, many later studies [12, 33, 34] that employed atomic absorption spectrophotometry did obtain higher copper levels in OSF patients. Anuradha and Devi also found lower levels of copper in plasma of OSF participants [27]. The sample size was 22, which was lower compared to most of the other studies. The method of Cu estimation was diethyldithiocarbamate followed by reading the results with a calorimeter. In addition, the patients included in this study had poor dietary patterns and experienced loss of appetite, which could have affected the Cu levels. Both these investigations were the earliest that compared levels of trace elements in OSF versus healthy participants. Other research studies have documented higher copper levels in oral mucosal tissue [4, 32], saliva [28], but not in the serum as compared to healthy controls. It has been suggested that the alterations in the levels of trace elements are seen predominantly in oral tissues and may not manifest themselves systematically in the serum and plasma [65]. Many of the other later studies, however, did document higher levels of Cu in the serum of OSF patients [23, 29, 30]. The biomarkers (saliva/serum/plasma) in the OSF group were used as moderators. The subgroup analysis indicated that the biological fluid did not have a significant influence on the effect size (*p* > 0.05). In other words, the levels of Zn, Cu, and Fe did not vary significantly because of the biological fluid they were measured from. The statistically insignificant effect of the type of biological fluid used could be due to the smaller number of investigations that measured trace elements in the saliva.

Iron serves many vital functions in the body. It is required for the transport of oxygen from the lungs to the cells in the form of hemoglobin, plays an important role in DNA synthesis and energy metabolism, and is crucial for the development and maintenance of oral mucosa. In investigations that have evaluated the levels of Fe in OSF [23, 27, 30, 36, 38, 52, 54, 56, 60], mean serum Fe concentrations in OSF patients were lower than the control groups, and the collagen content was significantly higher.

It has been suggested that diminution in Fe levels in OSF might be due to excessive utilization of Fe in the hydroxylation of proline and lysine for collagen synthesis [93]. Additionally, the chewing areca nut induces mechanical injury to the oral tissues, which may hamper the ability to ingest a nutritionally adequate diet [94]. Finally, chronic iron deficiency is a risk factor for the development of OSF [94] and anemia has been noted in the advanced stages of OSF [94].

The role of Fe in carcinogenesis remains controversial, as both excess and deficiency of iron have been linked to increased risk of developing cancer [95]. A chronic deficiency of iron may increase the susceptibility of the oral mucosa to the irritants from the areca nut [25]. Iron deficiency also has been associated with epithelial abnormalities and tumors of the mouth and pharynx [96]. The key features of OSF, chronic inflammation and epithelial dysfunction, have been observed in individuals and lab animals with iron deficiency [95]. Chronic inflammation has been associated with greater risk of cancer in many organs of the body [97]. Additionally, deficiency of iron may increase oxidative stress and DNA damage, conditions that have been linked to carcinogenesis [95]. Again, there is not enough evidence on release of Fe from its enzymes in OSF, but Winrow et al. have suggested that under acidotic conditions of the inflammation, iron is released from ferritin and xanthine oxidoreductase, leading to increase in Fe-catalyzed free radicals [98]. Paul et al. suggested that increase in iron content in the tissues of OSF patients could be because of similar inflammatory condition generated in OSF [45]. However, most of the studies recorded lower Fe in OSF patients; hence, additional research is needed to document the release of Fe from its enzymes in OSF patients. Thakur and Guttikonda [60] found that both hemoglobin and serum ferritin levels were reduced, while total iron binding was increased, in OSF patients compared to healthy controls. Similarly, Rajendran et al. [49] and Anuradha and Devi [27] also documented increased iron binding capacity in OSF versus healthy controls.

Few studies have documented the effect of supplementation of antioxidants and micronutrients therapy in correction of nutritional deficiencies in OSF patients. Thakur et al. found a significant improvement in the mouth opening at the end of 6 weeks in patients who were supplemented with the Mmo3 capsule [99]. Maher et al. documented significant improvement in burning sensation and mouth opening for 117 participants that were supplemented with a combination of vitamins (A, B complex, C, D, and E) and minerals (iron, calcium, copper, zinc, and magnesium) [100]. Jirge et al. observed improvement in the mouth opening by 6.7% in the OSF participants who received antioxidants (beta carotene: 10 mg; zinc sulphate monohydrate: 27.5 mg; selenium dioxide: 70 mg; manganese: 2 mg; copper: 1 mg) for 15 weeks [101]. Borle and Borle reported healing of vesicles, improvement in burning sensation, and relaxation in stiffness of the buccal mucosa folds in patients who were given Vitamin A, oral ferrous fumarate, and betamethasone for 3 weeks [102]. However, none of these studies reported the levels of micronutrients after intervention. Proteolytic enzymes such as hyaluronidase [103] and chymotrypsin also have been used in management of OSF [104]. However, none of these studies measured the levels of trace elements in OSF patients after intervention or after therapy. Therefore, no concrete data are available on the possible recovery of Fe, Cu, and Zn to normal levels after treatment with chelators or other medications. Since the present research is a meta-analysis that statistically summarized the results from others, data could not be derived on the recovery of Fe, Cu, and Zn.

## 5. Limitations

A limitation of this research is the relatively small sample size of many of the studies included in this meta-analysis. Also, different measurement techniques and biological specimens (serum, plasma, and saliva) were utilized for assessment of levels of trace elements, resulting in substantial between-study heterogeneity. This heterogeneity is shown by the high I^2^ value of 97.24%. Although it cannot be determined with certainty that trace element levels are different between OSF and healthy controls, most studies found statistically significant differences in trace element levels of Zn, Cu, and Fe between OSF patients and healthy controls.

Dietary intake data were not reported in the studies included; thus, the influence of diet is unknown. Yet, it has been observed that restricted mouth opening in OSF participants can predispose one to dietary deficiencies.

It is recognized that serum ferritin and transferrin receptors are the biomarkers of choice for measuring Fe status [105], but the current study was restricted to use of serum/plasma/salivary levels of Fe. However, one parameter of Fe status, hemoglobin, has been reported to be lower in OSF subjects [24, 27, 42–44, 106]. Recently, Thakur et al. [60] found that both hemoglobin and serum ferritin levels were reduced, while total iron binding was increased, in OSF patients compared to healthy controls. Similarly, Rajendran et al. [49] and Anuradha and Devi [27] also documented increased iron-binding capacity in OSF versus healthy controls. Thus, future research should include other biomarkers of iron status.

## 6. Conclusions

This is the first meta-analysis that has investigated the variations in the status of trace elements in the saliva/plasma/serum of patients suffering from OSF, as compared to healthy individuals by the use of the statistical technique of robust variance estimation. The use of this technique accounted for the dependency between these micronutrients. Also, this method allowed for pooling the studies together to calculate a pooled effect size for Zn, Cu, and Fe, controlling for the variations in laboratory methods and biological samples used by different investigators. In addition, this research included calculations of the ratios of Zn/Cu and Cu/Fe from these studies and conducted a meta-analysis to evaluate differences in these ratios between OSF and healthy participants. Levels of Zn and Fe were lower, and Cu levels higher, in OSF patients as compared to controls. OSF is a potentially malignant lesion that can cause significant morbidity and mortality. Cessation of areca nut chewing may be the first most important step towards reducing the occurrence and progression of this disease. Previous studies have investigated the effectiveness of micronutrient supplementation in resolving the symptoms of OSF. OSF patients have been supplemented with Cu in two studies. This is important to consider as this meta-analysis reported higher levels of Cu in OSMF patients as compared to controls. It is plausible that Cu, in higher concentrations, could aggravate any oxidative damage. Additional research is needed to investigate the potential adverse health effects of copper supplementation in OSF patients.

## Figures and Tables

**Table 1:** Characteristics and moderators of studies included in the meta-analysis investigating differences in levels of zinc, copper, and iron between healthy controls and subjects with oral submucous fibrosis.

Author, year (reference)	Biomarker	Age	Men (%)	Sample size (*n*)		Effect size		
				Patient	Control	Zinc	Copper	Iron
Gupta et al., 1987 [12]	Serum	35	65	40	10	0.112	1.264	—^b^
Varghese et al., 1987 [50]	Serum	38.85	16	50	50	−3.800	−6.541	—^b^
Rajendran et al., 1992 [49]	Serum	—^a^	—^a^	50	50	—^b^	—^b^	−30.170
Anuradha and Devi, 1995 [27]	Plasma	—^a^	—^a^	22	22	−5.436	−5.730	−8.461
Luqumun et al., 2003 [50]	Serum	17–40	67	15	15	—^b^	1.846	−0.349
Pillai and Burde, 2005 [32]	Serum	23.40	—^a^	40	31	—^b^	−0.427	—^b^
Khanna and Karjodkar, 2006 [52]	Serum	25–70	83	30	30	—^b^	0.862	−3.156
Nayak et al., 2010 [65]	Serum	22–60	95	20	20	−0.345	−0.404	—^b^
Balpande and Sathawane, 2010 [23]	Serum	—^a^	—^a^	30	30	−2.386	4.572	−8.975
Shettar, 2010 [31]	Serum	29.60	80	30	30	−1.806	0.975	—^b^
Tadakamadla et al., 2011 [33]	Serum	18–70	90	50	50	—^b^	1.145	−0.579
Ayinamudi and Narsimhan, 2012 [63]	Serum	23–62	67	15	6	0.959	1.683	—^b^
Shetty et al., 2012 [53]	Serum	—^a^	—^a^	65	21	—^b^	—^b^	−0.452
Shetty et al., 2012 [53]	Saliva	—^a^	—^a^	65	21	—^b^	—^b^	−0.399
Hegde et al., 2012 [66]	Serum	26.85	85	15	60	—^b^	—^b^	−3.501
Neethi et al., 2013 [55]	Serum	36.53	73	30	30	−0.521	0.758	—^b^
Khanna et al., 2013 [34]	Serum	36.85	—^a^	30	30	0.216	0.809	—^b^
Kode and Karjodkar, 2013 [28]	Serum	—^a^	—^a^	30	15	1.424	0.205	−0.642
Kode and Karjodkar, 2013 [28]	Saliva	—^a^	—^a^	30	15	0.497	0.512	−0.517
Kapoora et al., 2013 [24]	Serum	15–55	84	50	50	−17.943	83.349	−37.786
Shetty et al., 2013 [54]	Serum	—^a^	94	50	50	−4.932	—^b^	−9.023
Hosthor et al., 2014 [29]	Serum	—^a^	—^a^	30	30	−4.612	6.336	−4.729
Gurprasad et al., 2014 [56]	Serum	32.5	90	50	35	—^b^	—^b^	−2.853
Shetty et al., 2015 [30]	Saliva	—^a^	—^a^	50	50	−2.485	10.989	−14.582
Yadav et al., 2015 [35]	Saliva	28.6	84	50	50	−1.112	1.644	—^b^
Okade et al., 2015 [36]	Saliva	24.3	97	30	30	—^b^	0.291	−1.862
Hafeez et al., 2015 [57]	Serum	49	88.8	89	89	—^b^	—^b^	−0.601
Saurabh et al., 2015 [58]	Serum	18–45	83.3	30	30	—^b^	—^b^	−3.901
Srilekha, 2015 [37]	Serum	30–50	60	22	22	−0.223	2.545	—^b^
Mohammed et al., 2016 [61]	Saliva	32.73	90	30	30	—^b^	1.772	—^b^
Kallalli et al., 2016 [59]	Serum	20–60	78	30	10	—^b^	—^b^	−7.002
Tiwari et al., 2016 [38]	Serum	41.1	75	40	30	—^b^	2.349	−2.074
More and Patel, 2016 [26]	Serum	32.43	73	30	30	−10.576	3.015	—^b^
Kumar et al., 2016 [64]	Serum	42.6	89	35	35	—^b^	0.927	−0.508
Bhardwaj et al., 2016 [62]	Serum	16–65	83	40	40	—^b^	—^b^	−2.085
Thakur and Guttikonda, 2017 [60]	Serum	—^a^	—^a^	40	40	—^b^	—^b^	−1.966

^a^Not reported in the primary study. ^b^Not measured in the primary study.

**Table 2:** Zn/Cu ratios calculated from different studies.

Reference	Biomarker	Controls	OSMF	Total sample	Difference in Zn/Cu (OSMF-control)
Gupta et al. [12]	Serum	0.87	0.72	50	−0.15
Varghese et al. [50]	Serum	0.92	0.85	100	−0.07
Nayak et al. [65]	Serum	0.88	0.92	40	0.04
Balpande and Sathawane [23]	Serum	1.72	1.09	60	−0.63
Ayinamudi and Narsimhan [63]	Serum	0.05	0.04	21	−0.01
Shettar [31]	Serum	0.96	0.76	60	−0.2
Neethi et al. [55]	Serum	0.73	0.7	60	−0.03
Khanna et al. [34]	Serum	1.47	1.32	60	−0.15
Kode and Karjodkar [28]	Serum	1.48	2.05	45	0.57
Kode and Karjodkar [28]	Saliva	13.23	14.7	45	1.47
Hosthor et al. [29]	Serum	0.77	0.19	60	−0.58
Shetty et al. [30]	Saliva	0.78	0.28	100	−0.5
Yadav et al. [35]	Saliva	1.19	0.47	100	−0.72
More and Patel [26]	Serum	2.92	0.86	60	−2.06

**Table 3:** Cu/Fe ratios calculated from studies included.

Reference	Biomarker	Controls	OSMF	Total sample	Difference in Zn/Cu (OSMF-control)
Anuradha and Devi [27]	Plasma	1.2	2	44	0.8
Luqumun et al. [51]	Serum	1.06	1.37	30	0.31
Khanna and Karjodkar [52]	Serum	1.11	1.26	60	0.15
Balpande and Sathawane [23]	Serum	1.22	2.09	60	0.87
Tadakmadla et al. [33]	Serum	0.88	1.16	100	0.28
Kode and Karjodkar [28]	Serum	0.99	1.36	45	0.37
Kode and Karjodkar [28]	Saliva	0.11	0.33	45	0.22
Hosthor et al. [29]	Serum	1.06	5.25	60	4.19
Shetty et al. [30]	Saliva	0.61	2.45	100	1.84
Okade et al. [36]	Saliva	0.1	1.75	60	1.65
Tiwari et al. [38]	Serum	0.99	1.25	70	0.26
Kumar et al. [64]	Serum	0.89	1.15	70	0.26

## Data Availability

The dataset used for this analysis lies with the corresponding author and can be made available on request.
